# Improving Employee Performance in Industrial Parks: An Empirical Case of Garment Enterprises in Binh Duong Province, Vietnam

**DOI:** 10.3390/ejihpe10010005

**Published:** 2019-07-24

**Authors:** Thanh-Lam Nguyen, Pham Xuan Giang

**Affiliations:** 1 Office of International Affairs, Lac Hong University, Dong Nai 810000, Vietnam; 2 Faculty of Business Administration, Industrial University of Ho Chi Minh City, Ho Chi Minh City 700000, Vietnam

**Keywords:** employee performance, job satisfaction, determinants, garment enterprises, industrial parks, Vietnam

## Abstract

In responding to the current international integration and fierce competition on marketplace, over the last few decades, most businesses have tried to continuously improve their performance for better competitiveness. One of the preferred approaches is to enhance their employee performance; thus, fully capturing its determinants is critical. Thus, this study aimed at identifying key factors affecting employee performance so that businesses can create proper policies and actions to improve their overall performance. Specifically, as a common phenomenon, most employees working in industrial parks not only live far away from their workplaces as well as work a lot of overtime. These issues were carefully considered in this study to investigate their impacts on the employee satisfaction and performance. In the empirical case of garment enterprises in Binh Duong industrial parks, it was found that job satisfaction and employee performance are positively affected by eight factors: (1) reward and recognition; (2) development and training; (3) job promotion; (4) income; (5) work environment; (6) relationship with superiors; (7) relationship with colleagues; and (8) work procedure and role. In addition, it was found to be negatively affected by the house–work distance and overtime work, which are two new factors proposed in this study.

## 1. Introduction

The current trend of regional and international integration has brought numerous opportunities and challenges to the survival and growth of every business due to the significant increase in fierce competition on the marketplace. Besides, the rapid advances in technologies and policies at both national and international levels require businesses to have good quality performance and quality improvement systems in place [1,2]. In addition, employee involvement has been well recognized for its importance towards quality performance and quality improvement, meaning that the involvement is critical to the total quality management strategy [3]. Therefore, over the last few decades, most businesses have made special efforts to improve their organizational performance by continuously enhancing their employee involvement and performance as well as job satisfaction since this leads to the sustainable quality improvement within the organization. Lyons [4] found that it is critical to have organizational objectives understood by all employees and such objectives well aligned with the workforce skills, competency requirements, development plans and the delivery of results. As such, providing appropriate training opportunities to improve employee performance is of great importance in building a high performance workforce to successfully achieve the overall business strategy. Effective and efficient employee performance usually results in positive organizational performance [5]; as a consequence, employing proper management skills and schemes to improve the employee performance, efficiency and productivity is becoming more and more important these days [6].

With the rising competition from other provinces, especially Ho Chi Minh City and Dong Nai, Binh Duong should pay more attention to improve their workforce satisfaction and performance for its sustainable development in the context of international and regional integration. This study aimed at identifying the determinants of the employee performance so that some managerial implications could be proposed to help the local authorities and manufacturing enterprises in directing their feasible policies to improve the workforce performance in the province. An empirical case of garment enterprises in Binh Duong industrial parks was analyzed as a typical example to demonstrate its applicability.

## 2. Literature Review

### 2.1. Employee Performance

Several definitions of “employee performance” have been proposed; for instance, Hatane [7] defined employee performance as the contribution of an employee to the total output of an organization while Cascio [8] considered it as the accomplishment of the tasks assigned. Among them, this study employed the one proposed by Motowidlo [9] who defined it as “the total expected value to the organization of discrete behavioral episodes that an individual carries out over a standard period of time” because it fits our research scope.

Over the past few decades, employee performance has been considered as the strategic key for the survival and development of every organization in the recent competitive marketplace. Kohli et al. [10] claimed the positive effects of the investment in enhancing employee capability towards the improvement of employee performance while Zahargier and Balasudaram [11] indicated that employee performance is a critical factor directly affecting the outcome of positive behavior improvement and the increase in the organizational productivity.

Employee performance can be successfully measured with acceptable criteria that are established and agreed as their standards. According to Blickle et al. [12], employee performance consists of three core elements: (1) task performance to conduct assigned duties in terms of effectiveness and efficiency; (2) contextual performance to support the organizational social and psychological environment, which has indirect contribution to organizational performance; and (3) adaptive performance to deal with any sudden or unanticipated events happened during conducting their tasks or the organization. Consequently, employee performance has a positive influence on the overall performance of any organization [13]. Thus, improving the employee performance is critical to the increase of organizational performance.

### 2.2. Factors Affecting Job Satisfaction and Employee Performance

To successfully improve the employee performance, it is important identify the key factors affecting employee performance. Specifically, the following determinants have been discussed in the literature.

#### 2.2.1. Income

Rynes et al. [14] claimed that income plays important roles in motivating employees to improve their performance and their productivity. Income is also a critical determinant of employee satisfaction [15] and the retention of high quality personnel [16,17,18].

#### 2.2.2. Job Promotion

There is a positive relationship between job promotion and performance among employees [19,20,21]; hence, a mechanism for timely recognizing and promoting high performance employees helps not only to motivate them to work better but also to make them satisfied with their efforts [22,23,24,25].

#### 2.2.3. Work Environment

Work environment has significant impacts on employee satisfaction [26,27,28,29]. Specifically, physical settings, facilities and equipment, internal communication, group norms and values, employee engagement, leadership style, supports from senor leaders, etc. should be carefully considered and arranged in such a way as to improve employee’s behavior, attitude to work and productivity [30,31].

#### 2.2.4. Relationship with Superiors

Several scholars such as Russell-Bennett et al. [20], Nelson and Quick [18], and Rothwell and Kazanas [32] have well affirmed that mutual relationships and understanding between superiors and employees are important in motivating employees to improve their work performance, and be engaged and loyal to the organization. Besides, such relationship also significantly affects employee satisfaction and retention [33]. Therefore, it is encouraged that senior leaders should know how to stimulate employees to perform their tasks with inspiring words and motivational approaches [34,35].

#### 2.2.5. Relationship with Colleagues

A good relationship among employees is always an important issue in the human resource management activities because employees are then willing to support each other and it helps to avoid envy, impediments, staff doubt and bad rumors, and increase their solidarity to achieve the organizational performance [36]. Moreover, with such strong teamwork relationship, employees tend to be fully engaged and stay committed to their organizations [37,38] and satisfied with their jobs [31,39,40,41].

#### 2.2.6. Procedure and Role

Organizational procedures, roles and directives have significant impacts on the job satisfaction [42,43] and employee performance; specifically, if they are clear, employees can easily perform their tasks in a correct way, and their performance and the overall performance of the organization is improved accordingly [44].

#### 2.2.7. Reward and Recognition

A proper recognition mechanism plays a critical role in improving job satisfaction [45,46], employee performance, productivity and commitment [22,47]. Reio and Callahon [48] claimed that attractive awards and motivational approaches in recognizing good performance help employees to be more productive, effective and efficient.

#### 2.2.8. Development and Training

Development and training is one of the key functions in human resource management because it helps not only to enhance employee capability, morale and performance but also to keep pace with the advances in science and technology and achieve organizational goals [49,50,51,52] and job satisfaction [53,54].

#### 2.2.9. Job Security

Job security is usually referred to as the certainty level of still having one’s current job in the future. Low security means high risk of losing one’s job; hence, it is one of the key determinants of job satisfaction [55,56] and personal performance. Specifically, once feeling secure in their job, employees tend to be more satisfied and devoted to their work [57,58,59].

#### 2.2.10. Job Satisfaction

Job satisfaction is usually defined as the feeling of employees on doing their jobs in relation to their past experience, current context and expectations. There is a positive relationship between job satisfaction and employee productivity, commitment and retention [60] as well as the organizational performance [60,61,62].

## 3. Research Method

Based on the above identified factors, qualitative interviews with nine experts working as directors, managers or team leaders in two garment enterprises located in an industrial park in Binh Duong Province as well as four group discussions with 24 employees were conducted to discover the appropriateness of the listed factors and other potential ones. Through this qualitative research, the above-mentioned factors were found suitable to be further considered; besides, two other factors were added: (1) overtime working, which directly affects the health and mental focus of the employees; and (2) house–work distance, which directly affects the amount of time an employ spends traveling from their house to work place and their health as well. According to the literature review presented in Section 2 and the two newly proposed factors, this study investigated the hypotheses stated in Table 1.

**Figure 1:**
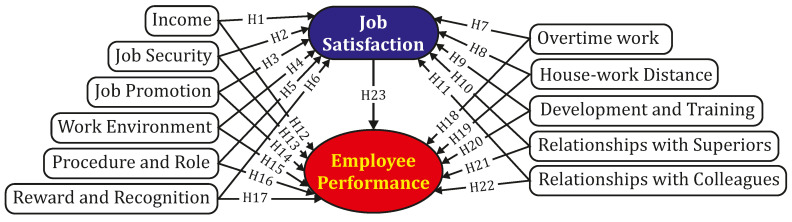
Proposed research model.

Therefore, the research model used in this study is presented in Figure 1.

Consequently, we created a survey questionnaire of 11 independent and 2 dependent constructs, as shown in Table 2. Each investigated items in the 11 independent constructs was stated in a positive manner and we asked the participants to provide the level of their agreement on each item based on a five-point Likert scale where 1 indicates “Strongly disagree” and 5 indicates “Strongly agree”. For example, under the construct “Development and training”, one of the four observed variables is “Opportunity in taking training courses is fairly offered to employees”. Meanwhile, with each item in the dependent construct, we asked them to self evaluate the level of their current satisfaction level as well as their performance also based on a five-point Likert scale where 1 indicates “Totally dissatisfied”/“Far below my ability” and 5 indicates “Totally satisfied”/“With my utmost ability”. For example, under the construct “Job satisfaction”, one of the four observed variables is “The physical working environment”. A pilot test with 12 employees was conducted to clarify the meaning and word usage. The refined version containing 51 items is briefly coded in Table 2. For brevity, the full version of the questionnaire will be provided on request.

To have better understanding of the relationships among these factors, data from these valid observations were used for our further analyses and tests, including scale reliability with Cronbach’s Alpha coefficient, exploratory factor analysis (EFA), confirmatory factor analysis (CFA) and structural equation modeling (SEM) with the help of a computational software SPSS V20.0.

Nunnally and Bernstein [63] claimed that a scale is considered reliable if its observed variables result in a corrected item-total correlation greater than 0.3 and a Cronbach’s Alpha coefficient greater than 0.7. Meanwhile, Hair et al. [64] proposed a set of evaluation criteria used in EFA, including: (1) eigenvalue ≥ 1; (2) total variance explained ≥ 50%; (3) Kaiser-Meyer-Olkin Measure (KMO) ≥ 0.5; (4) Significance level (Sig.) coefficient of the KMO test ≥ 0.05; (5) factor loadings of all observed variables are ≥0.5; and (6) weight difference between the loadings of two factors 0.3. The CFA was used to further confirm the unidirectionality, scale reliability, convergence value and distinctive value while SEM was used to test the fitness of our proposed model. According to Steenkamp and Trijp [65] and Hair et al. [66], a model is considered suitable for market data if the significance value of Chi-square test is no more than 5%; CMIN/df ≤ 2 (in some cases, CMIN/df ≤ 3 is also acceptable); and TLI and CFI ≥ 0.9. Besides these criteria, recent researchers suggest that GFI should be greater than 0.8, RMSEA ≤ 0.08, overall reliability should be greater than 0.6, and the extracted variance should be greater than 0.5 [66].

## 4. Empirical Results

Of the 365 hard copies of the refined questionnaire directly delivered to 31 garment enterprises located in different industrial parks in Binh Duong Province, 297 copies were collected. Among them, 34 were invalid, thus only 263 were valid, accounting for 72% of the total delivered. Table 3 briefly presents the demographic characteristics of the participants.

### 4.1. Exploratory Factor Analysis and Scale Reliability Analysis

First, we conducted exploratory factor analysis for the 43 items in the 11 independent factors and another analysis for 8 items in the two dependent ones. The KMO value of 0.873 and the significance of Bartlett’s Test of Sphericity of 0.000 demonstrated in Table 4 indicate that using EFA in this study is appropriate. Moreover, eigenvalues for these 11 components are all greater than 1.052 and these factors account for 66.18% of the total variance, showing that these scale items are unidimensional. Table 5 demonstrates the factor loadings of extracted factors and relevant results of scale reliability tests. The Cronbach’s Alpha coefficients of the factors are all larger than 0.79, indicating that the items in the factors have high internal consistency. Furthermore, as all of the corrected item-total correlations are greater than 0.3, these 11 factors are considered good enough and reliable for further analysis.

In the same token, the other 8 items used to measure the two dependent scales “Job satisfaction” and “Employee performance” were also analysed with EFA, which resulted in KMO = 0.865 with the significance level 0.000 shown in Table 6. These figures indicate that using EFA in this study is appropriate. Moreover, eigenvalues for these two components are all greater than 1.273 and these factors account for 74.5% of the total variance, showing that these scale items are unidimensional. Table 7 demonstrates the factor loadings of extracted factors and relevant results of scale reliability tests. The Cronbach’s Alpha coefficients of the factors are all larger than 0.80, indicating that the items in the factors have high internal consistency. Furthermore, as all of the corrected item-total correlations are greater than 0.3, these two scales are considered good enough and reliable for further analysis.

### 4.2. Confirmatory Factor Analysis

**Figure 2:**
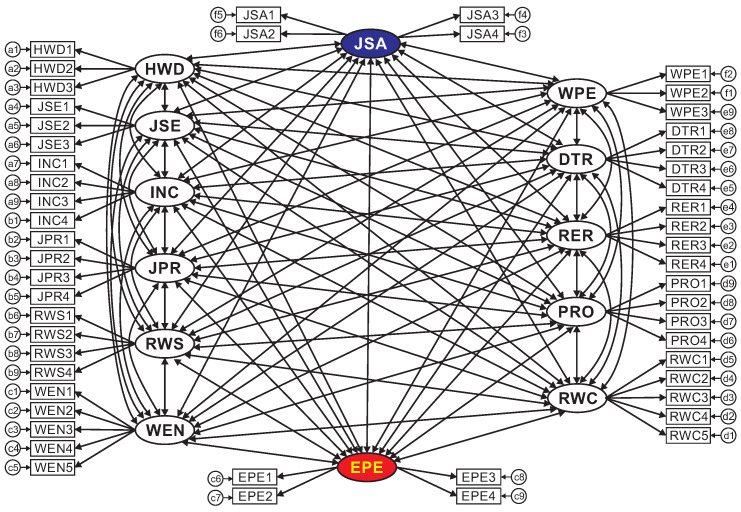
CFA results of the saturated model.

Table 8 briefly presents the test results of composite reliability and extracted variance of the factors affecting the performance of employees in garment enterprises in Dong Nai Province, where we can conclude that the investigated scales are reliable and consistent for further analysis. Figure 2 shows the results of the saturated model in CFA, including: Chi-squared = 1806.857, df = 1146, Chi-squared/df = 1.577 2, *p*-value 0.1%, TLI = 0.928, CFI = 0.935 and RMSEA = 0.047. These parameters well satisfy the required criteria for CFA, meaning that the investigated elements in the proposed model are unidirectional, convergent, reliable and distinctive. Therefore, we can conclude that the research model is consistent with the actual data. Table 9 presents the full correlation coefficients mentioned in Figure 2 for clarity.

### 4.3. Structural Equation Modeling

#### 4.3.1. Model of Job Satisfaction and Employee Performance

**Figure 3:**
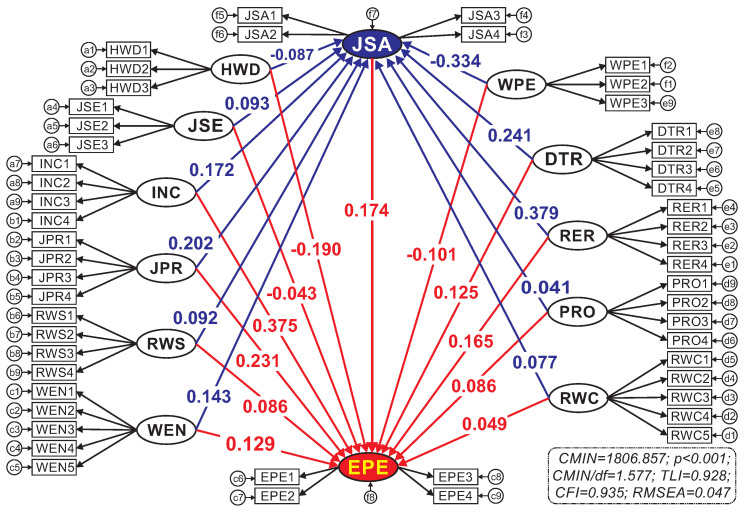
Standardized SEM model.

After the EFA and CFA analyses as presented above, SEM analysis was used to identify the determinants of job satisfaction and employee performance. Figure 3 briefly shows the analysis results where CMIN = 1806.857, CMIN/df = 1.577 2, *p*-value 0.001, TLI = 0.928, CFI = 0.935 and RMSEA = 0.047, which also well satisfy the required evaluation criteria for the SEM model. Thus, it is concluded that the model is consistent with the actual data. Moreover, by using bootstrap approach with 2000 times, the results shown in Table 10 indicate that the bias of the model is insignificant because the abstract values of the critical ratios are all less than 1.96, cumulative normal distribution at the significance level of 5%, indicating that the estimates of the model shown in Figure 3 are reliable.

#### 4.3.2. Hypothesis Tests Using the SEM Model

The results of the model estimation and bootstrapping in the SEM shown in Table 10 clearly indicate that the obtained regression coefficients (except those of JSE → JSA and JSE → EPE) are statistically significant as their respective *p*-values are less than 0.05. Consequently, 21 out of 23 stated hypotheses (except H2 and H13) are supported.

## 5. Discussion and Conclusions

### 5.1. Discussion

As shown in Table 11, job satisfaction was found to be positively affected by eight factors, which also further agree with some previous findings mentioned in Section 2. Specifically, the eight factors include: (1) reward and recognition [22,47,48]; (2) development and training [49,50,51,52]; (3) job promotion [19,20,21,22,23,24,25]; (4) income [14,15,16,17,18]; (5) work environment [26,27,28,29]; (6) relationship with superiors [18,20,32,33,34,35]; (7) relationship with colleagues [36,37,38]; and (8) work procedure and role [44]. However, job satisfaction is negatively affected by the house–work distance and the overtime work, which are newly considered in this study. Between them, the overtime work was found more negative because they usually feel exhausted if they are frequently requested to take overtime work. Although overtime work may help the employees to significantly increase their income, they fail to have free time to take care of their families and enjoy their lives as well as take some development courses to enhance their competence. In addition, the long distance between their house to the workplace also negatively affects their job satisfaction. It was found that many employees in the garment enterprises working in Binh Duong industrial parks live in different places and far away from their workplaces. Some ride motorbikes themselves while some are picked up by the company buses. To get to work on time, they need to get up early in the morning and get back late in the evening; gradually, they become tired and sometime get sick with the travelling daily.

Moreover, this study found that reward and recognition (β = 0.379) plays the most important role in the job satisfaction of the employees in the garment enterprises while income (β = 0.172) is ranked in fourth place. It is because their monthly income is almost fixed and acceptable compared to other companies in the industrial parks. From our practical investigation, the rewards help them to improve their income and working motivation. Furthermore, the insignificant difference of the monthly income among the enterprises fails to retain the employees; in addition, there is a high demand of working labor in the industrial parks, thus they can easily switch their jobs. That is why the job security was found insignificant in this study. Therefore, to make the employees more satisfied, a good mechanism for rewards, awards and recognition of individual and team contribution should be carefully considered and improved. The development and training (β = 0.241) as well as job promotion (β = 0.202), respectively, come in second and third. Along with the reward and recognition mechanism, the enterprises need to provide training programs and/or supportive policies for employees to enhance their competence and get promoted. Such actions will positively improve employees’ satisfaction. It is also worth noting that work environment (β = 0.143) also has significant impact on job satisfaction.

Besides, these factors also significantly affect the overall performance of the employees. It was found that income, job promotion, job satisfaction and reward and recognition are the most important factors contributing to their performance. Work environment and the development and training programs are also critical to improve the employee performance. This finding further emphasizes the importance of the mentioned proposals, which will make them not only satisfied but also perform better.

Importantly, the two newly proposed factors, overtime work and house–work distance, were found to negatively affect the employee satisfaction and performance. These factors have been neglected in several previous studies. Therefore, the garment enterprises and/or related industrial parks are suggested to offer good dormitory or some local housing services so that their employees can save their time and improve their health. For those who cannot stay in the dormitory or use the local services due to personal issues, the enterprises may offer some alternative options such as faster transportation, better buses, etc. to improve their satisfaction and accordingly enhance their performance.

Lastly, our practical investigation shows that the simplicity and routine of related activities in their daily work results in the lowest impacts of the “work procedure and role” on the employee satisfaction and performance.

### 5.2. Conclusions

The recent changes in the business context, especially the fierce competition on the marketplace, require every business to continuously improve themselves for their own survival and growth. Furthermore, improving employee performance is one of their preferred approaches to gain their competitive advantages. Hence, all business organizations are making a special effort to fully identify the determinants of employee performance so that they can create proper policies and actions to improve their performance. The research objectives of this study well aligned with the practical demand. Specifically, in the empirical case of garment enterprises in Binh Duong industrial parks, through common analyses such as scale reliability analysis, exploratory factor analysis, confirmatory factor analysis and structural equation modeling, it was found that job satisfaction is positively affected by eight factors: (1) reward and recognition; (2) development and training; (3) job promotion; (4) income; (5) work environment; (6) relationship with superiors; (7) relationship with colleagues; and (8) work procedure and role. In addition, it is negatively affected by the house–work distance and overtime work, which are two new factors proposed in this study. Besides these ten factors, employee performance was also found to be influenced by the job satisfaction.

This study had some limitations, such as research space and the sample size of the participants. As this research was limited to the garment enterprises in Binh Duong industrial parks, future research would enlarge the research space by investigating more garment enterprises in other industrial parks in southern Vietnam or investigating the difference among different industries in Binh Duong industrial parks. Moreover, the insignificant coefficients of JSE indicate that further investigation on the impacts of job security to job satisfaction and employee performance should be conducted.

## Figures and Tables

**Table 1:** Research hypotheses.

Hypotheses		Expectation
H1	Income has positive impacts on job satisfaction.	+
H2	Job security has positive impacts on job satisfaction.	+
H3	Job promotion has positive impacts on job satisfaction.	+
H4	Work environment has positive impacts on job satisfaction.	+
H5	Work procedure and role has positive impacts on job satisfaction.	+
H6	Reward and recognition has positive impacts on job satisfaction.	+
H7	Overtime work has negative impacts on job satisfaction.	−
H8	House–work distance has negative impacts on job satisfaction.	−
H9	Development and training has positive impacts on job satisfaction.	+
H10	Relationship with superiors has positive impacts on job satisfaction.	+
H11	Relationship with colleagues has positive impacts on job satisfaction.	+
H12	Income has positive impacts on employee performance.	+
H13	Job security has positive impacts on employee performance.	+
H14	Job promotion has positive impacts on employee performance.	+
H15	Work environment has positive impacts on employee performance.	+
H16	Work procedure and role has positive impacts on employee performance.	+
H17	Reward and recognition has positive impacts on employee performance.	+
H18	Overtime work has negative impacts on employee performance.	−
H19	House–work distance has negative impacts on employee performance.	−
H20	Development and training has positive impacts on employee performance.	+
H21	Relationship with superiors has positive impacts on employee performance.	+
H22	Relationship with colleagues has positive impacts on employee performance.	+
H23	Job satisfaction has positive impacts on employee performance.	+

**Table 2:** Coding of the latent and observed variables.

No.	Construct	Code	No. of Items	Item Codes
1	Income	INC	4	INC1 → INC4
2	Job Promotion	JPR	4	JPR1 → JPR4
3	Work Environment	WEN	5	WEN1 → WEN5
4	Relationship with Superiors	RWS	4	RWS1 → RWS4
5	Relationship with Colleagues	RWC	5	RWC1 → RWC5
6	Procedure and Role	PRO	4	PRO1 → PRO4
7	Reward and Recognition	RER	4	RER1 → RER4
8	Development and Training	DTR	4	DTR1 → DTR4
9	Job Security	JSE	3	JSE1 → JSE3
10	Overtime work	WPE	3	WPE1 → WPE3
11	House–work Distance	HWD	3	HWD1 → HWD3
12	Job Satisfaction	JSA	4	JSA1 → JSA4
13	Employee Performance	EPE	4	EPE1 → EPE4

**Table 3:** Descriptive statistics of participants.

Characteristics		No. of Observations	Percentage (%)
Gender	Male	108	41.06
	Female	155	58.94
Age	30	126	47.91
	30–40	84	31.94
	40–50	34	12.93
	=50	19	7.22
Education	Under high school	47	17.87
	High school	136	51.71
	Bachelor	66	25.1
	Postgraduate	14	5.32

**Table 4:** KMO and Bartlett’s Test of independent variables.

Kaiser–Meyer–Olkin Measure of Sampling Adequacy		0.873
Bartlett’s Test of Sphericity	Approx. Chi-square	7131.693
	df	903
	Sig	0.000

**Table 5:** Results of EFA analysis and scale reliability analysis.

	Factor											α	Corrected Item-Total Correlation	α If Item Deleted
	1	2	3	4	5	6	7	8	9	10	11			
WEN4	0.97											0.896	0.757	0.871
WEN2	0.77												0.736	0.875
WEN3	0.71												0.709	0.881
WEN5	0.69												0.752	0.872
WEN1	0.68												0.768	0.868
RWC3		0.82										0.860	0.693	0.827
RWC5		0.78											0.714	0.822
RWC4		0.71											0.688	0.828
RWC2		0.65											0.605	0.849
RWC1		0.65											0.688	0.829
INC4			0.81									0.881	0.693	0.866
INC3			0.81										0.697	0.865
INC1			0.80										0.846	0.806
INC2			0.76										0.737	0.850
RWS4				0.88								0.872	0.758	0.824
RWS3				0.82									0.732	0.834
RWS1				0.79									0.778	0.816
RWS2				0.67									0.640	0.870
DTR1					0.87							0.893	0.808	0.844
DTR3					0.82								0.754	0.865
DTR2					0.81								0.752	0.866
DTR4					0.79								0.736	0.871
PRO1						0.91						0.891	0.831	0.832
PRO3						0.80							0.741	0.867
PRO2						0.80							0.738	0.868
PRO4						0.78							0.732	0.870
RER1							0.89					0.882	0.778	0.836
RER4							0.84						0.771	0.839
RER3							0.75						0.726	0.856
RER2							0.72						0.704	0.864
JPR4								0.88				0.888	0.776	0.848
JPR3								0.83					0.790	0.842
JPR1								0.61					0.742	0.861
JPR2								0.56					0.710	0.872
JSE1									0.92			0.885	0.823	0.795
JSE2									0.84				0.762	0.849
JSE3									0.79				0.744	0.865
WPE1										0.85		0.799	0.705	0.659
WPE2										0.72			0.609	0.761
WPE3										0.72			0.618	0.752
HWD1											0.88	0.795	0.711	0.639
HWD2											0.70		0.598	0.761
HWD3											0.69		0.606	0.753

Extraction method: principal axis factoring; rotation method: Promax with Kaiser normalization.

**Table 6:** KMO and Bartlett’s Test of dependent variables.

Kaiser–Meyer–Olkin Measure of Sampling Adequacy		0.865
Bartlett’s Test of Sphericity	Approx. Chi-square	2902.372
	df	208
	Sig	0.000

**Table 7:** Results of EFA analysis and scale reliability analysis.

Construct		Factor		α	Corrected Item-Total Correlation	α If Item Deleted
		1	2			
Employee performance	EPE1	0.887		0.891	0.892	0.883
	EPE2	0.881			0.875	0.888
	EPE3	0.861			0.862	0.891
	EPE4	0.843			0.874	0.888
Job satisfaction	JSA3		0.75	0.812	0.631	0.764
	JSA4		0.725		0.614	0.772
	JSA1		0.715		0.685	0.737
	JSA2		0.633		0.593	0.782

Extraction method: principal axis factoring; rotation method: Promax with Kaiser normalization.

**Table 8:** Confirmatory factor analysis.

Term	Construct	No. of Observed Variables	Reliability Test	
			Cronbach’s Alpha	Composite Reliability
Factors affecting job satisfaction and employee performance	Income (INC)	4	0.881	0.874
	Job Promotion (JPR)	4	0.888	0.888
	Work Environment (WEN)	5	0.896	0.896
	Relationship with Superiors (RWS)	4	0.872	0.873
	Relationship with Colleagues (RWC)	5	0.860	0.861
	Procedure and Role (PRO)	4	0.891	0.892
	Reward and Recognition (RER)	4	0.882	0.883
	Development and Training (DTR)	4	0.893	0.893
	Job Security (JSE)	3	0.885	0.887
	Overtime work (WPE)	3	0.799	0.800
	House–work Distance (HWD)	3	0.795	0.799
Job Satisfaction (JSA)		4	0.812	0.811
Employee Performance (EPE)		4	0.891	0.891

**Table 9:** Correlation coefficients between the constructs in Figure 2.

Correlation		Correlation		Correlation		Correlation	
JSA↔HWD	−0.049	PRO↔EPE	0.103	JSE↔WEN	−0.115	RWC↔JSA	0.545
JSE↔HWD	0.034	RER↔RWC	0.587	INC↔EPE	0.663	PRO↔HWD	−0.018
JSE↔INC	−0.05	DTR↔PRO	0.014	JPR↔RWC	0.574	JSE↔RER	−0.099
INC↔JPR	0.536	WPE↔RER	−0.036	PRO↔RWS	0.009	DTR↔INC	0.045
JPR↔RWS	0.483	DTR↔JSA	0.225	RER↔WEN	0.705	WPE↔JPR	0.009
RWS↔WEN	0.405	JSE↔JSA	0.049	DTR↔EPE	0.153	RWS↔JSA	0.468
WEN↔EPE	0.63	JPR↔HWD	0.02	WPE↔RWC	−0.094	EPE↔HWD	−0.145
RWC↔EPE	0.562	JSE↔RWS	0.029	PRO↔JSA	0.081	JSE↔RWC	0.082
PRO↔RWC	−0.046	INC↔WEN	0.679	RER↔HWD	0.017	INC↔PRO	0.009
RER↔PRO	0.067	JPR↔EPE	0.627	JSE↔DTR	0.041	RER↔JPR	0.726
DTR↔RER	−0.02	RWS↔RWC	0.586	WPE↔INC	−0.081	DTR↔RWS	0.196
WPE↔DTR	−0.031	PRO↔WEN	−0.003	JPR↔JSA	0.585	WPE↔WEN	−0.018
WPE↔JSA	−0.319	RER↔EPE	0.647	WEN↔HWD	−0.029	JSA↔EPE	0.657
WPE↔HWD	0.004	DTR↔RWC	0.141	JSE↔EPE	−0.053	RWC↔HWD	0.027
INC↔HWD	0.029	WPE↔PRO	−0.072	INC↔RWC	0.602	JSE↔PRO	0.05
JSE↔JPR	0.045	RER↔JSA	0.639	JPR↔PRO	0.036	INC↔RER	0.652
INC↔RWS	0.424	DTR↔HWD	0.047	RER↔RWS	0.454	DTR↔JPR	0.019
JPR↔WEN	0.686	WPE↔JSE	−0.006	DTR↔WEN	−0.042	WPE↔RWS	−0.127
RWS↔EPE	0.469	INC↔JSA	0.561	WPE↔EPE	−0.18	WEN↔JSA	0.559
RWC↔WEN	0.655	RWS↔HWD	0.068				

**Table 10:** Confirmatory factor analysis.

Relationship	Estimate	Bias	SE-Bias	Critical Ratio
JSA←WEN	0.143	−0.004	0.0037	−1.08
JSA←RWC	0.077	0.001	0.0020	0.50
JSA←RWS	0.092	−0.006	0.0056	−1.07
JSA←PRO	0.041	0.004	0.0039	1.03
JSA←JPR	0.202	−0.005	0.0053	−0.94
JSA←INC	0.172	−0.003	0.0018	−1.67
JSA←JSE	0.093	−0.002	0.0022	−0.91
JSA←HWD	−0.087	0.003	0.0022	1.36
JSA←RER	0.379	−0.009	0.0089	−1.01
JSA←DTR	0.241	−0.007	0.0101	−0.69
JSA←WPE	−0.334	0.009	0.0067	1.34
EPE←JSA	0.174	0.001	0.0025	0.40
EPE←RWC	0.049	0.001	0.0014	0.71
EPE←PRO	0.086	0.001	0.0008	1.25
EPE←RER	0.165	−0.003	0.0034	−0.88
EPE←DTR	0.125	−0.003	0.0040	−0.75
EPE←WPE	−0.101	0.003	0.0032	0.94
EPE←HWD	−0.190	0.006	0.0083	0.72
EPE←JSE	−0.043	0.002	0.0017	1.18
EPE←INC	0.375	−0.005	0.0155	−0.32
EPE←JPR	0.231	−0.004	0.0237	−0.17
EPE←RWS	0.086	−0.001	0.0089	−0.11
EPE←WEN	0.129	0.001	0.0010	1.00

**Table 11:** Confirmatory factor analysis.

Relationship	Unstandardized Coefficient	Standardized Coefficient β	S.E.	C.R.	*p*-Value	Conclusion
JSA←RER	0.285	0.379	0.109	2.615	0.009	H6 supported
JSA←DTR	0.145	0.241	0.035	4.143	***	H9 supported
JSA←JPR	0.143	0.202	0.070	2.043	0.0413	H3 supported
JSA←INC	0.129	0.172	0.066	1.955	0.0508	H1 supported
JSA←WEN	0.109	0.143	0.049	2.224	0.0263	H4 supported
JSA←JSE	0.044	0.093	0.041	1.073	0.2835	H2 rejected
JSA←RWS	0.053	0.092	0.020	2.650	0.0082	H10 supported
JSA←RWC	0.063	0.077	0.030	2.100	0.0359	H11 supported
JSA←PRO	0.018	0.041	0.007	2.571	0.0103	H5 supported
JSA←HWD	−0.052	−0.087	−0.020	2.600	0.0094	H8 supported
JSA←WPE	−0.145	−0.334	−0.035	4.143	***	H7 supported
EPE←INC	0.310	0.375	0.047	6.596	***	H12 supported
EPE←JPR	0.180	0.231	0.042	4.286	***	H14 supported
EPE←JSA	0.192	0.174	0.091	2.110	0.0351	H23 supported
EPE←RER	0.136	0.165	0.050	2.720	0.0066	H17 supported
EPE←WEN	0.108	0.129	0.048	2.250	0.0246	H15 supported
EPE←DTR	0.082	0.125	0.036	2.278	0.0229	H20 supported
EPE←PRO	0.041	0.086	0.020	2.050	0.0406	H16 supported
EPE←RWS	0.055	0.086	0.023	2.391	0.0170	H21 supported
EPE←RWC	0.045	0.049	0.017	2.647	0.0082	H22 supported
EPE←JSE	−0.022	−0.043	−0.016	1.375	0.1694	H13 rejected
EPE←WPE	−0.048	−0.101	−0.022	2.182	0.0293	H18 supported
EPE←HWD	−0.124	−0.190	−0.034	3.647	***	H19 supported

Notes: *** *p* 0.001.

## Abbreviations

The following abbreviations are used in this manuscript:

CFA	Confirmatory factor analysis
EFA	Exploratory factor analysis
KMO	Kaiser-Meyer-Olkin
SEM	Structural equation modeling
Sig.	Significance
